# Lead-free Single-molecule Switching Material with Electric, Optical, Thermal Triple Controllable Multifunction Based on Perovskite-like Crystal and Flexible Thin Film

**DOI:** 10.1038/s41598-017-12338-y

**Published:** 2017-10-02

**Authors:** Cheng Chen, Wan-Ying Zhang, Qiong Ye, Da-Wei Fu

**Affiliations:** Ordered Matter Science Research Center, Jiangsu Key Laboratory for Science and Applications of Molecular Ferroelectrics, Southeast University, Nanjing, 211189 P.R. China

## Abstract

With the flourishing development of star molecule (CH_3_NH_3_)PbI_3_, organic-inorganic perovskites with multifunction and flexibility have become a worldwide focus. However, the controllable photoelectric switchable material (especially electric, optical, thermal multifunctional switches) still face great challenges, and most of them are ceramic and toxic lead-based series. Herein a lead-free perovskite-like crystal and flexible thin film, ImMC (ImMC = (HIm)_6_∙[MnCl_4_∙MnCl_6_]) (1), with many advantages over inorganic ceramics and lead-based perovskites, performs ideal optical and dielectric duple switching properties simultaneously. The order-disordered HIm (Im = imidazole) cations of α-type occupy two lattice sites corresponding to “Switch-ON/0” and “Switch-OFF/1” states, respectively. Interestingly, the optical and dielectric “ON/OFF or 0/1” switches can be integrated into one single-molecule single/duple channel module with high signal-noise ratio, in which the “ON/OFF” response can be precisely controlled by temperature or/and light wavelength signal to realize automatically multiple switching. In brief, the lead-free multifunctional switch opens up a brand new route and shows the mark of its real genius as a highly desirable material for its advanced applications in highly integrated circuit and ultrahigh-encrypted storage in flexible photoelectric devices.

## Introduction

Intelligent controllable materials with outstanding electric, optical and thermal bistable versatility have attracted great attention for their tunable optoelectronic properties when applied in digital processing, luminescent lighting, storages, sensors, scintillator materials and optoelectronic technology, etc^1–6^. This advanced multi-functional feature will promote the application of multiple encryption devices, high-density memory devices and highly integrated devices in both military and civil fields^7–12^. But this kind of material is rarely reported, and most of them are environmentally toxic series, such as inorganic ceramics and lead-based perovskites. However, perovskite-type molecular materials possess prominent advantages over inorganic ceramics, such as energy-efficient, environmentally friendly, controllable, easy synthesis of thin films and large crystals, which are very conducive to the device fabrications and becoming the hotspot of current research^13–15^. For example, photovoltaic materials (CH_3_NH_3_PbI_3_)^16,17^ and lead-based white light-emitting perovskites (C_4_N_2_H_14_PbBr_4_)^18^ have shown great potential for their applications in field-effect transistors, light-emitting diodes, and photodetectors.

Therefore, we designed a new lead-free photoelectric switchable material (ImMC), which not only contains advantages of perovskite-type molecular materials such as CH_3_NH_3_PbI_3_, but also possesses excellent film-forming ability, high flexibility, lead-free and triple light-electric precise controllability, etc. It is found that HIm (Im = imidazole) cations exhibited good flexibility in molecular thermal motions, which usually plays the key role in induced factors^19–22^. What is more, as is well known that tetrahedral MnCl_4_ displays with green emission while octahedral MnCl_6_ displays with red. If the two types of coordination modes were assembled together, it would generate unusual physical properties. Recently, we have synthesized a large-size single crystal of compound ImMC (ImMC = (HIm)_6_∙[MnCl_4_∙MnCl_6_])^23^, which exhibits intense red luminescence under ultraviolet (UV) excitation and temperature-induced structure phase transition. After precise analyses of the basic structural unit, main packing and structure comparison, we investigated the phase transition mechanism schematics between the low temperature phase and the room temperature phase. It turns out that the translation movement of the HIm cations may be the driving force of the optical-electric transition. In addition, we have successfully designed the dense and transparent ImMC thin film by easier spin-coating process, which displays ideal flexibility, transparency and photoluminescence performance. Organic-inorganic hybrid materials always guarantee both the superior carrier mobility of inorganic semiconductors and the processability of organic materials^24–28^. This type of organic-inorganic hybrid thin film as the key component of optoelectronic devices possess prominent advantage of non-toxic, light weight, flexible and easy processing, which lays the foundation for broader application prospects.

## Results and Discussion

### Thermal properties, large-size single crystal and structural phase transition

**Figure 1:**
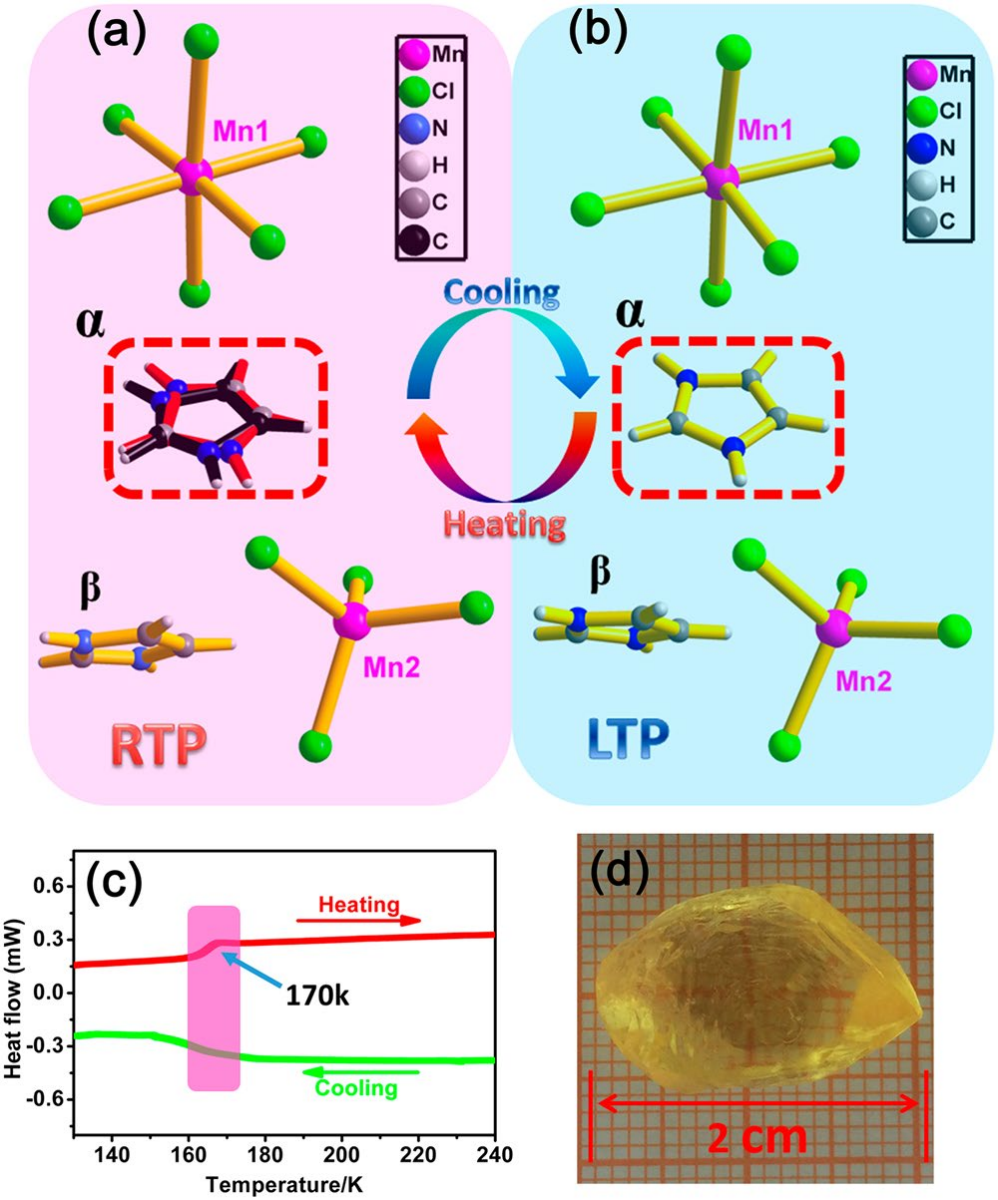
Viewing of four type discrete components in the basic structural unit and showing the coordination environment of compound 1 in RTP (**a**) and LTP (**b**). (**c**) DSC measurement of compound 1 obtained in heating-cooling mode. (**d**) The as-grown crystal of 1 with a size of 20 × 12 × 8 mm^3^.

Differential scanning calorimetry (DSC) measurement was used as the first evidence to verify the existence of reversible structural phase transition induced by temperature during the heating and cooling process. As shown in Fig. 1c, evident endothermic or exothermic efficiency induced curve fluctuations appeared at the phase transition temperature (170 K) with considerable peak width, which indicates that compound 1 undergoes a second-order phase transition. Entropy change ∆S in the heating process is approximately calculated as 4.39 J (K∙mol)^−1^. According to Boltzmann’s equation Δ*S* = *R* ln *N*, where *N* is the ratio of possible orientations and *R* is the gas constant. The *N* value of compound 1 is ca.1.69, coinciding fairly well with the DSC results and further confirming the typical order-disorder transition^29–32^.

In order to figure out the microscopic mechanism of phase transition, the structure details of compound 1 is revealed by the determination of variable-temperature crystal structures at 293 K (the room temperature phase, RTP) and 123 K (the low temperature phase, LTP), respectively (Fig. 1a and b). In the RTP, The basic unit consists of four type discrete components, i.e., tetrahedral (MnCl_4_)^2−^ anions, octahedral (MnCl_6_)^4−^ anions, planar HIm (α) cations and HIm (β) cations. We find that two different coordination modes of metal chloride coexist in one cell, which draws our attention to analyze it’s microstructure. Atom Mn1 is octahedrally coordinated by six Cl atoms, with Mn1−Cl distance of 2.5506(4) Å and 2.6050(7) Å for Mn1−Cl1 and Mn1−Cl2, respectively. Atom Mn2 is tetrahedrally coordinated by four Cl atoms, with an Mn−Cl distance of 2.3584(5) Å. One type of HIm cation shows evident disorder states, which is split into double imidazole rings. It means the disordered HIm (α) cations are moving between the two positions. Conversely, the HIm (β) cations always remain relatively static state in the room temperature. In the LTP, the basic unit consists of four type discrete components resembled with that of RTP. Atom Mn1 is octahedrally coordinated by six Cl atoms, with Mn1−Cl distance of 2.5402(13) Å and 2.5927(18) Å for Mn1−Cl1 and Mn1−Cl2. The atom Mn2 is tetrahedrally coordinated by four Cl atoms, with an Mn2−Cl distance of 2.3584(5) Å. However, different from RTP, both two HIm cations remain relatively stationary, indicating that HIm (α) cations undergo the process from disordered state to relative stable and ordered state.

**Figure 2:**
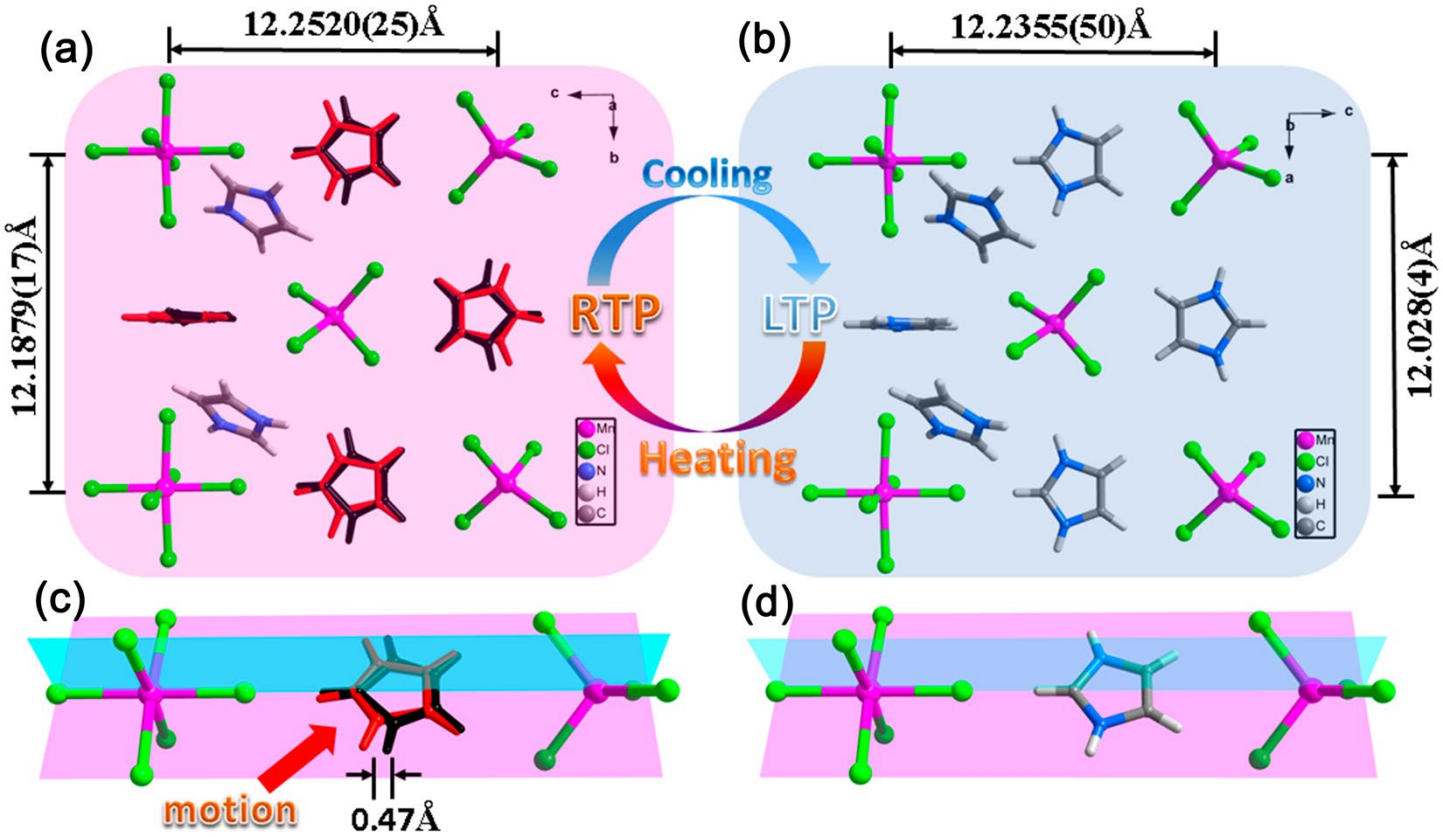
The view of RTP and LTP packing diagrams for 1 on the *bc* plane in RTP (**a**) and *ac* plane in LTP (**b**), combined with the phase transition mechanism schematics, which clearly show the motion of HIm cations in RTP (**c**) compared with static HIm cations in LTP (**d**).

In the packing diagrams, the compound 1 shows loosely double staggered array of tetrahedral (MnCl_4_)^2−^ anions and octahedral (MnCl_6_)^4−^ anions, when protonated HIm cations tightly fill in the space of these anions (Fig. 2a and b). The type of HIm (α) cations mainly fill in the space of tetrahedral (MnCl_4_)^2−^ anions, which display prominent disordered states in RTP. It seems that more loose space in tetrahedral (MnCl_4_)^2−^ anions contributes to the disordered motion of HIm (α) cations, while HIm (β) cations keep ordered states in the process of temperature changes (see Figure S3 in supporting information). Compared the packing diagrams of metal halides in the RTP with those in the LTP, the distance between tetrahedral Mn atoms and octahedral Mn atoms changes from 12.2520(25) Å to 12.2355(50) Å, and the distance of two tetrahedral Mn atoms (or two octahedral Mn atoms) changes from 12.1879(17) Å to 12.028(4) Å. These minor changes indicate that metallic framework keeps good stability in the phase transition process, which is consistent with the unchangeable space group of *I4(1)/a* before and after the phase transition. What is more, the disordered HIm (α) cations are split into two penta cyclics in RTP, indictive of a cationic displacive-type phase transition.When comparing the Fig. 2c and d, the 0.47 Å translation motion amplitude of HIm (α) cations in RTP become the major reasons of phase transition.

### Growth mechanism, morphology, unidirectionality and photoluminescence of flexible thin film

**Figure 3:**
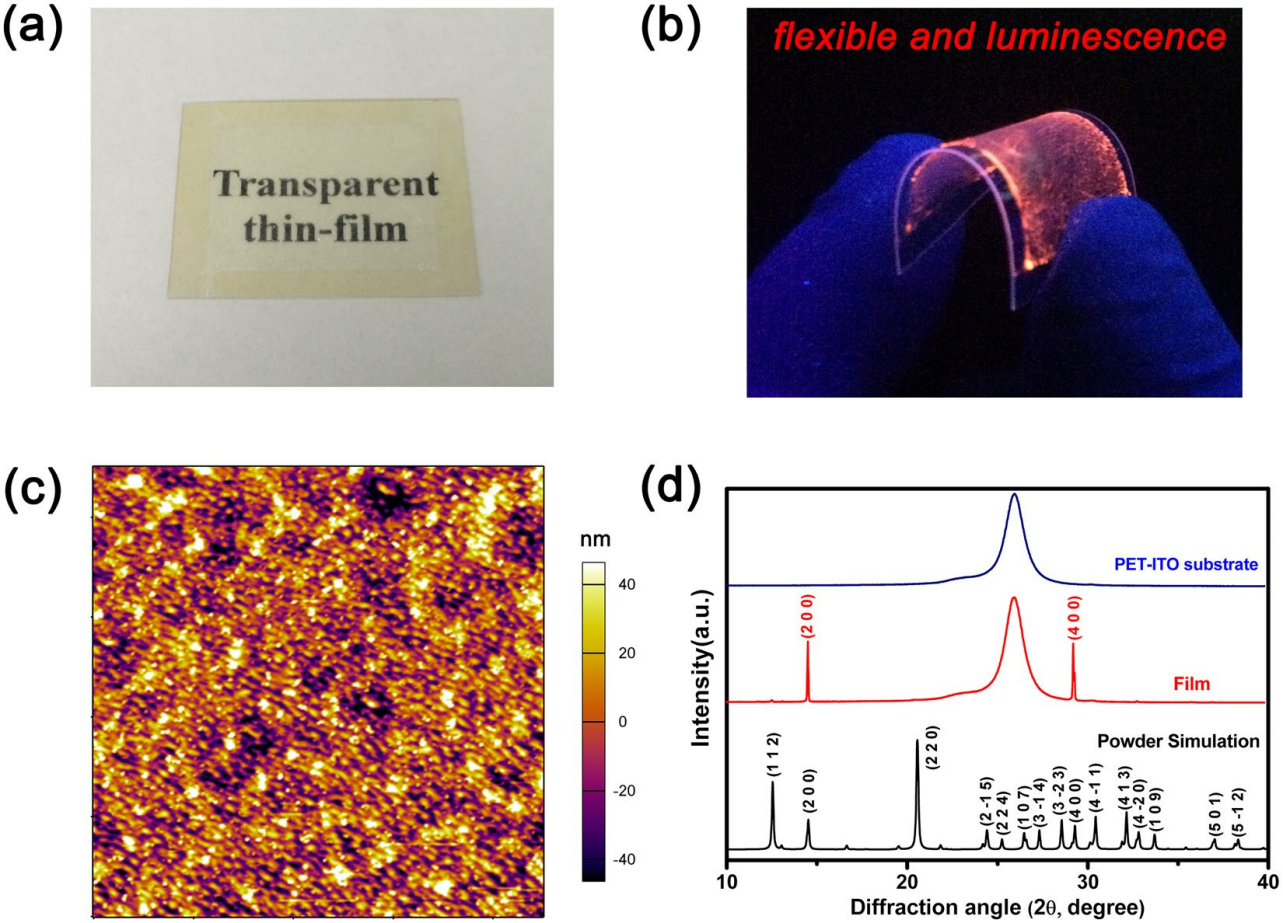
(**a**) Transparent thin film was fabricated on PET-ITO substrate plane by spin-coating method. (**b**) Favorable flexible and luminescence property of large bending of thin film under UV light. (**c**) Atomic force microscopy (AFM) images of the ImMC thin film displaying dense and uniform surface morphology. (**d**) XRD patterns of PET-ITO substrate and ImMC thin film fabricated on PET-ITO substrate, as well as powder simulation of compound 1.

Transparent thin-film devices formed on flexible substrates are expected to meet emerging technological demands while large block crystal materials cannot be applied in the integrated electronic devices. And the unique optical-electrical dual switching effect of the ImMC crystal motivated us to investigate thin films for easier miniaturization and integration, which were successfully prepared by the simple and inexpensive spin-coating approach^33–35^. As shown in Fig. 3a, the single-layered transparent thin film deposited on flexible PET-ITO substrates (PET: polyethylene terephthalate, ITO: indium tin oxide) by the spin-coating method, where we can see clearly that word “Transparent thin-film” on paper through the thin film sample. And Optical transmission spectrum of the ImMC thin film showed a high transmission of 80% or more in visible region (see Figure S7 in Supporting Information).Different from general perchlorate imidazole well-distributed dendritic crystal growth, which can finally merge into a seamless film as N,N-dimethylformamide (DMF) solvent continues to slowly evaporate^36,37^. The ImMC thin-film crystals were fabricated by rapid evaporation of methanol solvent in the spin-coating process, and uniform distribution of high concentrated methanol solution (solubility around 38%) contributed to the growth of transparent single-layer crystal in a very short period of time. The continuous ImMC single-layer crystals grew densely and uniformly on the PET-ITO substrate plate, which could be observed from the scanning electron microscopy (SEM) on the micrometer scale (see Figure S5 in Supporting Information). Atomic force microscopy (AFM) measurements were also implemented to capture the morphological images of ImMC thin film (Fig. 3c), and.the −40~40 nm film surface relief degree in a 90*90 μm^2^ range indicating that relatively smooth and dense thin-film morphology. Moreover, an AFM image (~200 nm) with ideal large magnification of thin-film surface morphology was provided in supporting information (Figure S6), which showed that the grains were well crystallized and homogeneously distributed. Here, morphology and compactness of well-distributed single-layer thin film are primarily decided by the solubility of saturated solution, spin-coating speed and follow-up treatments (ambient temperature). First, intense anisotropy in ImMC crystals and interface interaction between single-layer crystals and substrate jointly promote a preferred fast growth orientation. Second, suitable rotate speed ensure the continuous and uniformity of thin film, in case some large block crystal grain inlaid in thin film caused by slow evaporation of relatively high concentrated solution. We also tried to fabricate thin film by spreading saturated aqueous solution on substrate plane, and found that surface and thickness of the whole film are uneven without spin-coating process (see Figure S4 in Supporting Information). Third, the appropriate temperature to remove the residual solvent is necessary, and rapid high temperature drying solvent evaporation will produce a large number of bubbles to break the continuity of the thin film.

The X-ray diffraction tests were carried out on both ITO-PET substrate and thin-film sample at 293 K, and two evident diffraction peaks appeared at 14.56° and 29.32° corresponding to (200) and (400) crystal planes, respectively. No other reflections are detected in this experiment, which indicating that whole of thin-film crystal plane is unidirectional and along the direction of a-axis. Hence, the ImMC thin film is single crystalline-like structure and fabricating of this crystal thin film is subjected to the intense anisotropy. As shown in Fig. 3b, the bendable flexible thin film emitted strong red light similar to block crystal or powder, which further verifies the favorable single crystalline-like structure of thin film. The thickness of ImMC thin film is about 4 μm through measurement of a man-made gap with AFM, and excellent luminescent properties and anisotropy indicate that micron-sized thin films still retain the physical and chemical properties of the crystal. Moreover, the significantly curved thin film means that ImMC thin film has a good flexibility in manufacturing process and provides a basis for further miniaturization of the device.

### Dielectric switches, relaxation, anisotropy and Cole–Cole relation

**Figure 4:**
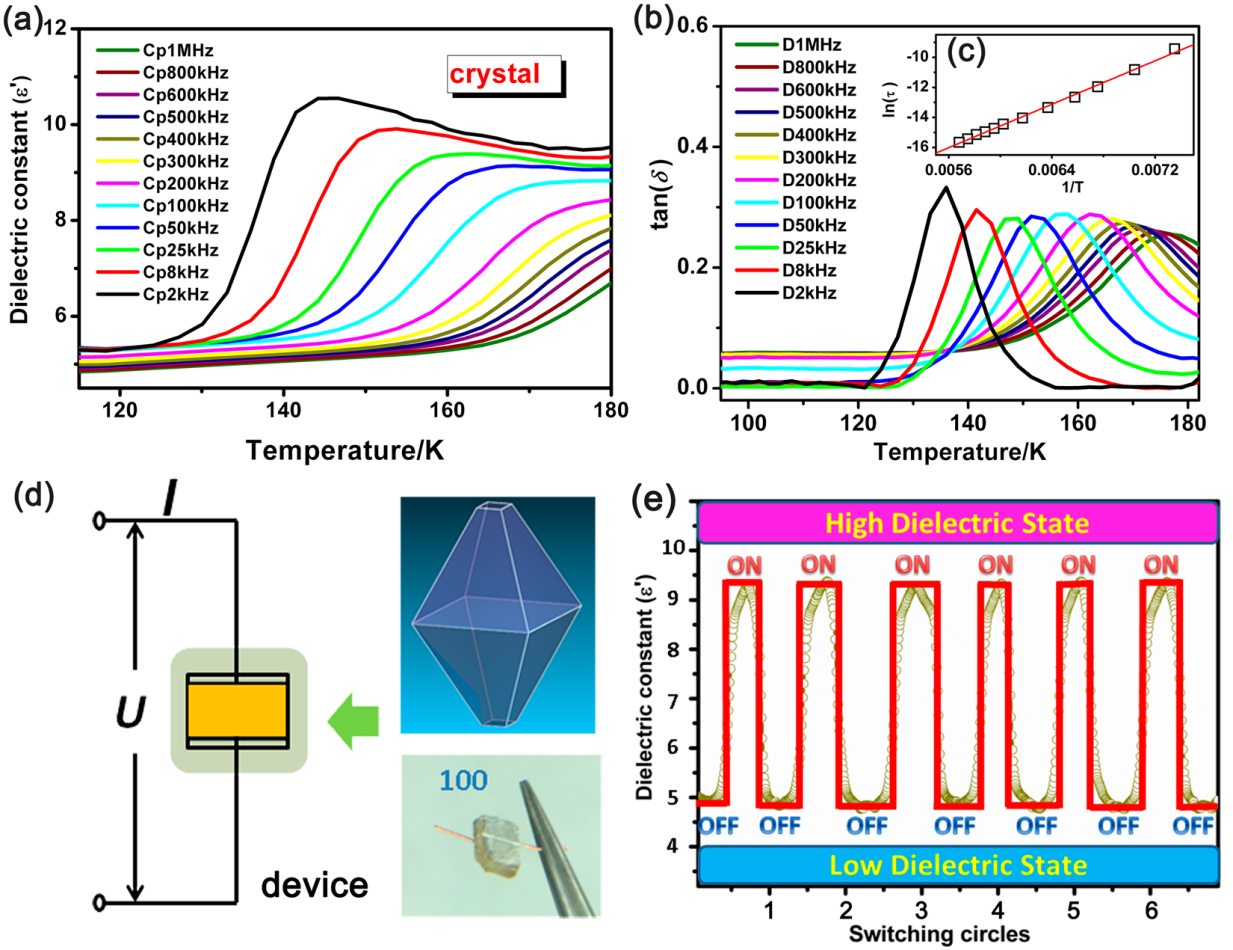
(**a**) Real part (*ε*′) of the dielectric permittivity of 1 measured along the a-axis at various frequencies in heating process. (**b**) Dielectric loss (tan *δ*) of the dielectric permittivity measured along the a-axis at various frequencies on heating. (**c**) Arrhenius plots for the dielectric relaxation on heating. (**d**) Viewing of the dielectric measurement mechanism schematic illustration and mono-crystalline electrode for compound 1 along the a-axis. (**e**) The recoverable switching circles of dielectric effects of compound 1 along the a-axis.

In Fig. 4a, the temperature-dependent dielectric permittivity *ε*′ (The real part (*ε*′) of the complex dielectric constant *ε* = *ε*′- i*ε*′′, where *ε*′′ is the imaginary part) of 1 was measured on single-crystal samples along the a-axis at various frequencies ranging from 2 KHz to1 MHz. Upon heating, the *ε*′ values for the crystal sample at various frequencies decrease continuously from 180 K to 130 K, accompanied by evident step-like switchable anomalies near phase transition temperature. As one kind of typical frequency-dependent materials, the permittivity at a lower frequency is somewhat larger than that at a higher frequency. Especially in the high frequency ca. 200 KHz, the accurate phase transition point (170 K) is in good accordance with the DSC results. Based on the structure analysis, the changes of *ε*′ values are ascribed to the molecular motions of HIm (α) cations. Generally, the cations get enough excitation thermal energy to be able to obey the change more easily in the external electric field^38–40^. In the RTP, the disordered HIm (α) cations achieve greater flexibility stimulated by the external electric field. The flexibility dramatically enhances their contribution to the polarization, finally leading to an increase in dielectric permittivity. Similarly, the freeze of HIm (α) cations in low temperature will weaken the flexibility and possess a weak contribution to the polarization. Further insight into the reason of such difference indicates that the a-axis is much more sensitive to the external excitation, especially at low frequency, which fits fairly well with switchable/tunable dielectric character.

Dielectric relaxation investigation is always used as a handle on molecular dynamics, and the temperature dependence of the permittivity at a constant frequency or its frequency dependence at a constant temperature acts as the principal types of measurement^41–43^. As shown in Fig. 4b, the dielectric relaxation behaviors of compound 1 can be explained by the peak maxima of tan *δ* changes obeying the Arrhenius equation *τ* = *τ*_0_ exp(*E*_a_/*k*_B_ *T*), where *τ*_0_ is the inverse of the frequency factor, *E*_a_ denotes the activation energy, *k*_B_ denotes the Boltzmann constant, and *T* is the temperature. For a Debye peak, the equation can be rewritten as ln *τ* = ln(2π*f*)^−1^ = ln(*τ*_0_) + *E*_a_/*k*_B_ *T*_p_, in which *f* is the frequency, and *T*_p_ is the temperature of the peak. From the experimental data of ln *τ* versus 1/*T* plot for the single-crystal sample along the a-axis (Fig. 4c, inset), *E*_a_ and *τ*_0_ can be approximately calculated to be 30.21 kJ mol^−1^ and 1.90 × 10^−16^ s, respectively. The magnitude of *E*_a_ is found to be large, taking into account a relatively small size of the HIm (α) cation. It indicates that HIm (α) cations contributing to the electric polarization of the crystal are strongly stiffened in the crystal lattice. Furthermore, as the frequency increases, the peak locates from 135 K to 180 K and becomes less pronounced. This can be explained by the fact that the charge carriers cannot follow the alternating electric field beyond the external field of a certain frequency. Such dielectric relaxation behaviors can be also observed in some other organic-inorganic compounds that undergo order−disorder type phase transitions^44–47^. Details of dielectric measurement mechanism schematic illustration and mono-crystalline electrode are shown in the Fig. 4d. The electrode were made by sputtering silver onto both sides (face 100) of single-crystal specimens and attaching copper leads with silver paste. What is more, dielectric constant (*ε*′) switching of crystal samples 1 is completely reversible between high dielectric state and low dielectric state (Fig. 4e). After several switching “ON”/“OFF” circles, the dielectric constant (*ε*′) values always remain unchanged and there is no value attenuation appeared in the measurement process. The dielectric constant (*ε*′) switching circles certainly demonstrate resistance to fatigue and sustainable utilization of crystal 1.

**Figure 5:**
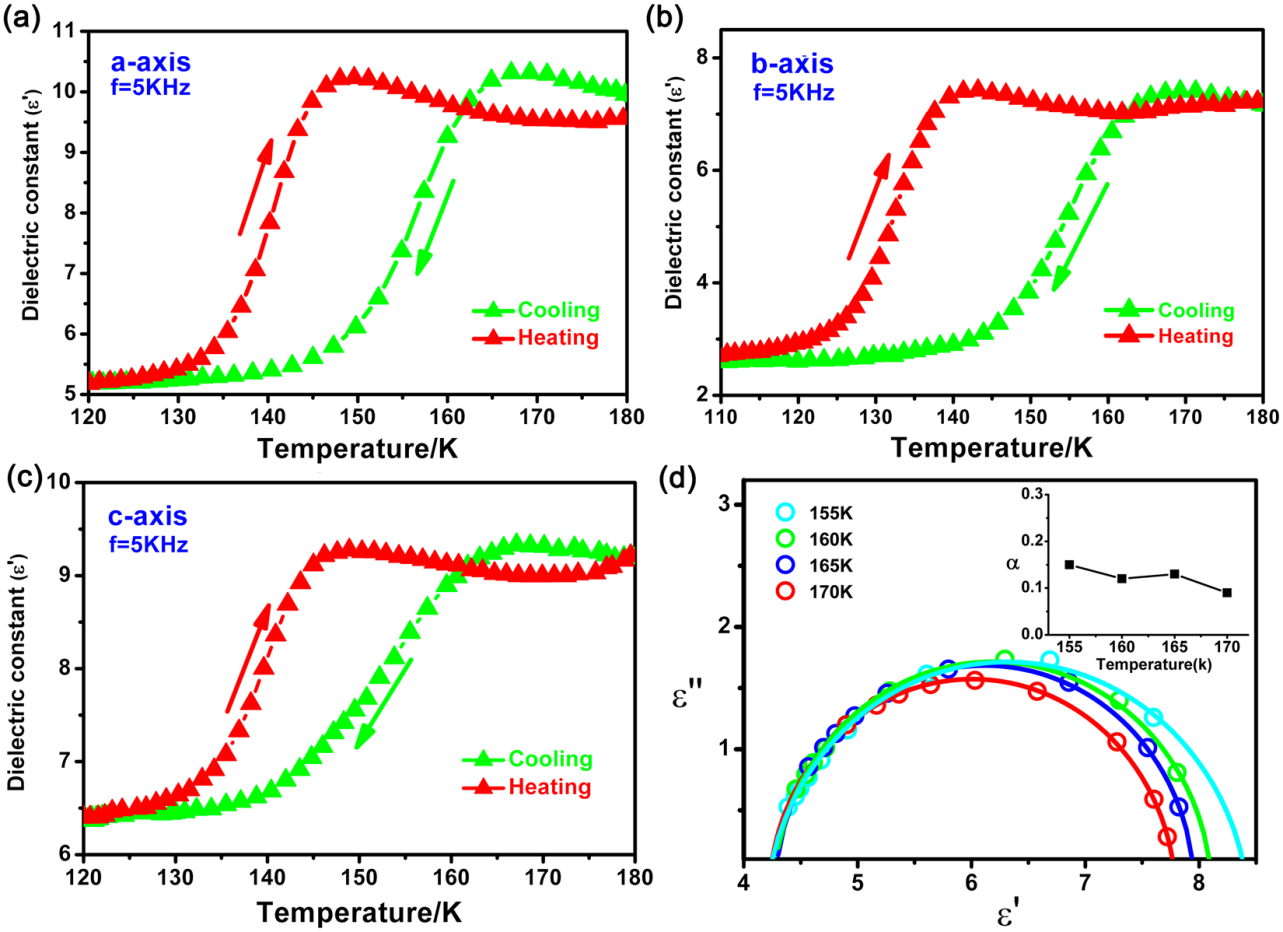
(**a**–**c**) Temperature-dependent dielectric permittivity (ε′) of compound 1 measured on crystal samples along the a-axis, b-axis, and c-axes at 5 kHz, respectively. (**d**) Cole–Cole plots of ε′′ versus ε′ at four selected temperatures (155 K, 160 K, 165 K, 170 K) showing relaxation nature of the dielectric dispersion in compound ImMC.

As shown in Fig. 5(a–c), we have performed measurements on anisotropic permittivity and found that compound 1 displayed evident anisotropy along the different directions of the a-axis, b-axis and c-axis at 5 kHz. With the temperature increasing/decreasing, a step-like permittivity increase/decrease along the three different crystallographic axes occurred at ca. 140 K/160 K, indicating that dielectric constant transition point moves to lower temperature at low frequency. On the a-axis, the value of real part (*ε*′) of the dielectric permittivity increases progressively from 5.2 to a maximal value of 10.2, when the *ε*′ changes from 2.8 to 7.2 along the b-axis and 6.4 to 9.2 along the c-axis. The smallest change value △*ε*′ = 2.8 appears on the c-axis comparing with a-axis (△*ε*′ = 5) and b-axis (△*ε*′ = 4.4). It can be explained by the crystal structure that the planar HIm (α) cations lie in the ac/bc crystallographic plane perpendicular to the a/b axis in the phase transition process, and the order-disorder induced HIm (α) cations dipolar changes always along a/b axis. Thus, the dynamic behaviors of the dipolar HIm (α) cations upon temperature changes yield less dipole-moment component on the c-axis.

It is interesting to find that the dielectric response of compound 1 in the relaxation process is well described by the Cole–Cole relation near phase transition temperature:

$$\varepsilon ={\varepsilon }_{\infty }+\frac{{\varepsilon }_{0}-{\varepsilon }_{\infty }}{1+{({\rm{i}}{\rm{\omega }}{\rm{\tau }})}^{1-\alpha }}$$

where *ε*_0_ and *ε*_∞_ are the low and high frequency limits of the electric permittivity, respectively, *ω* is the angular frequency, *τ* is the macroscopic relaxation time, and *α* is the distribution of the relaxation times parameter. As presented in Fig. 5d, the Cole–Cole diagrams for the single-crystal sample along a-axis at four selected temperatures (155 K, 160 K, 165 K, 170 K) deviate from semi-circles, which means we deal with a polydispersive relaxation process in compound 1. The empirical parameter *α* is fluctuating with temperature changes, which suggests that the distributions of the relaxation times depend on temperature changes and the observed dielectric relaxation processes are a kind of polydispersive character^48,49^.

### Photoluminescent switches from crystalline powders to large block single crystal

**Figure 6:**
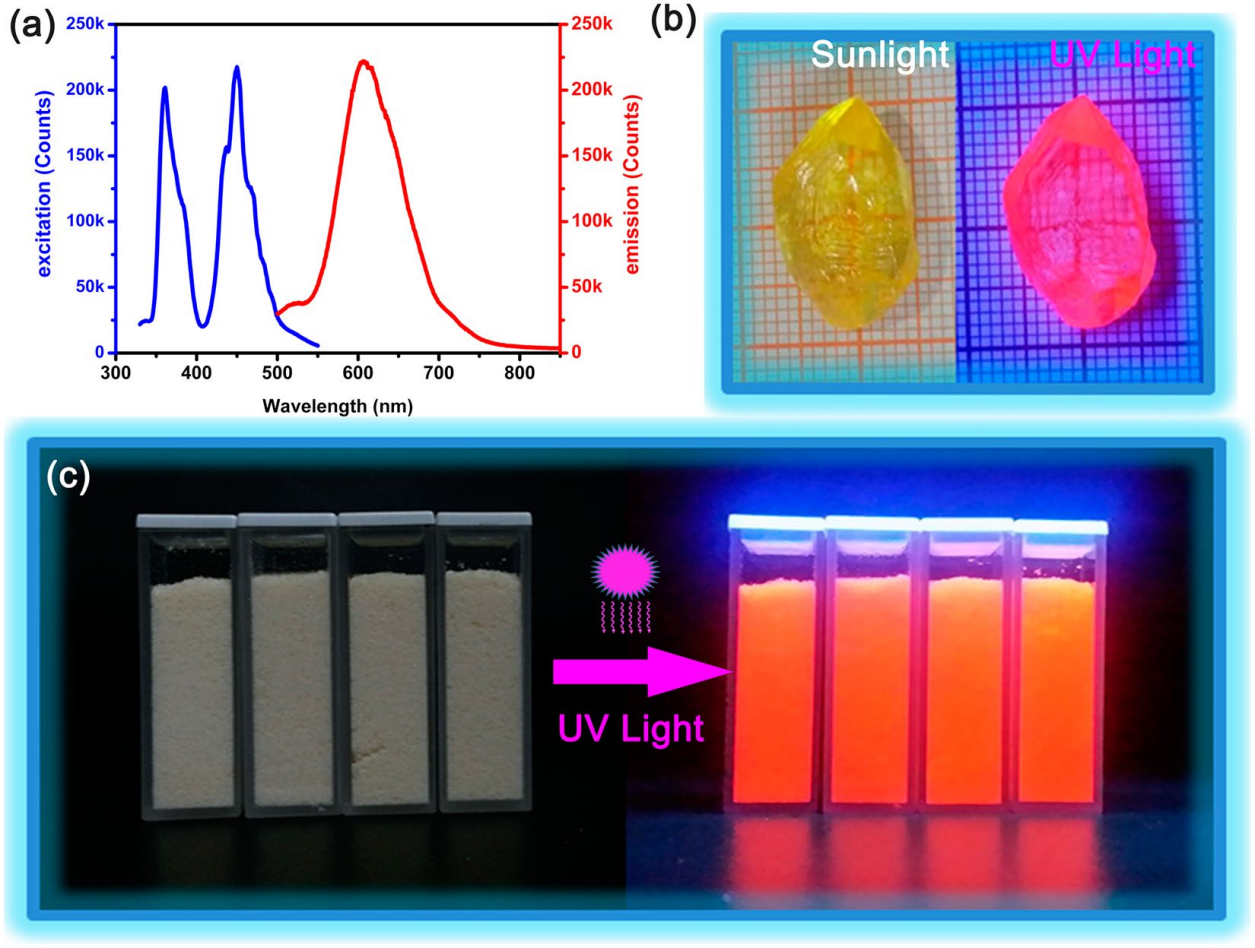
(**a**) Excitation and emission spectra of compound 1 at room temperature. (**b**) Yellow block crystal of compound 1 under sunlight and UV light, respectively. (**c**) Yellow powders of compound 1 put in four quartz cuvette and emit strong red light under UV light in the darkroom.

According to the previous research results, the position of the Mn^2+^ emission band depends on its coordination environment^50,51^. The Mn^2+^ ion is coordinated tetrahedrally with chlorine leading to green emission while the Mn^2+^ ion is coordinated octahedrally with chlorine contributing to red emission, which indicate that different surroundings of Mn^2+^ ions lead to different visible emissions. Compound 1 consists of both discrete MnC1_6_^4−^ octahedra and MnC1_4_^2−^ tetrahedra which are separated by the HIm cations and exhibits unique photo-luminescence properties. When illuminated with a UV lamp (365 nm), the crystals and powders emit strong red light (Fig. 6b and c). The excitation spectrum of the powder sample includes two sharp peaks from visible to the ultraviolet region (Fig. 6a). One sharp peak (450 nm) owing to^6^A_1_g(S) −^4^T_2_g(G) transition of Mn^2+^ ions can be observed at the visible region (400–550 nm) in the excitation spectrum. And the other one shows strong excitation line peaking at 360 nm in the UV region, which corresponds to the^6^A_1_g(S)−^4^Eg(D) transition of Mn^2+^ ions. While in the emission spectrum, when the excitation wavelength is 450 nm, a strong emission can be observed at 606 nm (FWHM is approximately 90 nm). Such interesting photoluminescence is attributed to the emission originating from the^4^T_1_g(G)−^6^A_1_g(S) transition of Mn^2+^ ions^52,53^. What’s more, the crystal fields of tetrahedral complexes are generally much weaker than octahedral complexes so that both absorption and emission should occur at longer wavelength, which provide a good explanation for the red photoluminescence of compound 1.

**Figure 7:**
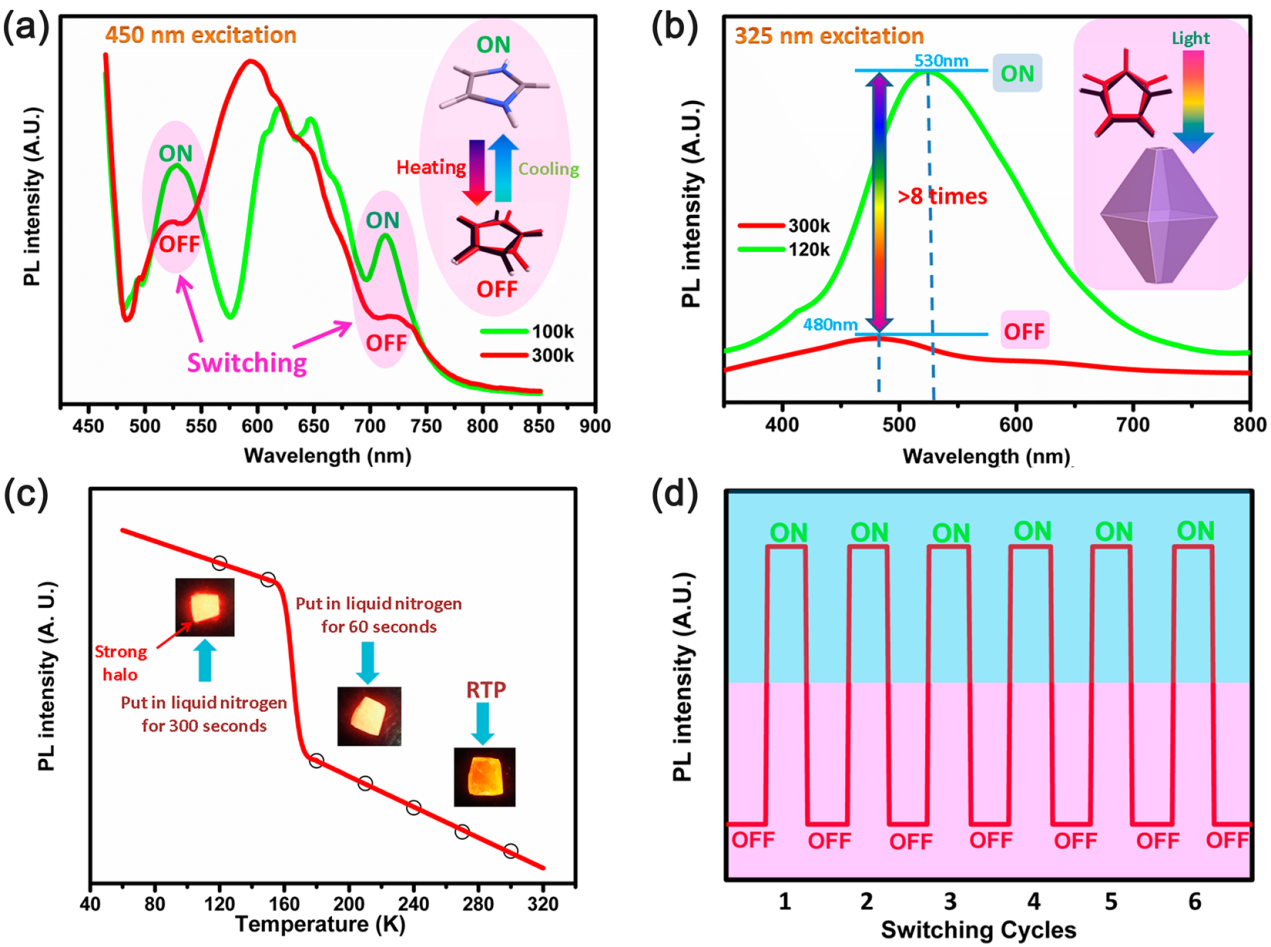
(**a**) Emission spectra of powder sample 1 at 100 K and 300 K under 450 nm excitation, luminescent intensity switching “ON”/“OFF” process accompany with structure phase transition. (**b**) In order to achieve single-peak luminescent intensity switching “ON”/“OFF” effect, we adopted 325 nm wavelength excitation on block crystal at 120 K (switch “ON” for low temperature) and 300 K (switch “OFF” for high temperature). (**c**) Peak value changes of luminescent intensity from block crystal 1 in cooling process. (**d**) Schematic diagram of luminescent intensity switching “ON”/“OFF” cycles.

The temperature-induced structural phase transition also caused novel molecular optical (fluorescence) switch characteristics, which are rarely reported. As shown in Fig. 7a, the emission spectrum of powder sample displaying evident changes between RTP (300 K) and LTP (100 K). In RTP, only one evident emission peak (600 nm) and two weak inflection points (528 nm and 713 nm) can be observed. When the temperature decreases to the LTP, the two inflection points translate to two obvious emission peaks (528 nm and 713 nm). In this process, unique temperature-depended switching effect is activated between switch “OFF” states in RTP and switch “ON” states in LTP. The switching changes can be attributable to structural phase transition of HIm (α) cations moving. Energy loss of thermal motion of the disordered HIm (α) cations lead to weaker intensity of the emission spectrum in RTP. With the decrease of temperature, HIm (α) cations freezing in ordered states reach to switch “ON” states in LTP.

In order to obtain “clear” single switchable effect and avoid multipoint response interference, we try to change excitation wavelength instead of fixed 450 nm wavelength excitation. After several tries, 325 nm wavelength light source was selected as the excitation light source. In addition, the large block crystal sample is also designed to test Photoluminescence characters. As shown in Fig. 7b, After 325 excitation on one block crystal, the emission spectra at 300 K and 120 K are recorded. Different from powder samples, only one weak emission peak can be observed at 480 mm in room temperature (300 K) and one relatively strong peak at 530 nm in the low temperature (120 K). The intensity of the emission spectra at 120 K is 8 times higher more than 300 K, indicate that the intensity of the emission spectrum changed accompany with the occurrence of the structural phase transition. The changes show evident bistable properties in the two phases (the low emission peak intensity represents switch “OFF” in high temperature, and the high emission peak intensity indicates switch “ON” in low temperature). This bistable properties is further confirmed by peak value changes of luminescent intensity in the cooling process as show in Fig. 7c, and abrupt increases of peak values at around 170 K is consistent with structure phase transition. What’s more, the obvious luminescence changes of block crystal can be observed under UV lamp (365 nm) in the cooling process (inset in Fig. 7c). And more strong halo around block crystal appear in LTP (switch “ON” state) comparing with weak halo of crystal in RTP (switch “OFF” state). The luminescence changes of block crystal are exactly synchronized with changing trends of peak values, indicating the pretty optical switch effect of compound 1. Besides, luminescent intensity switching of crystal samples 1 is completely reversible as shown in Schematic diagram of Fig. 7d. After several switching “ON”/“OFF” circles, the luminescent intensity values can remain stable and no value attenuation appear in the measurement process, which is consistent with dielectric constant (*ε*′) switching process. The perfect reversible of luminescent intensity switching and dielectric constant (*ε*′) switching further demonstrate optical-electrical synchronous switch effect of crystal 1.

### Electric, optical, thermal triple controllable and multifunctional integration modularity

**Figure 8:**
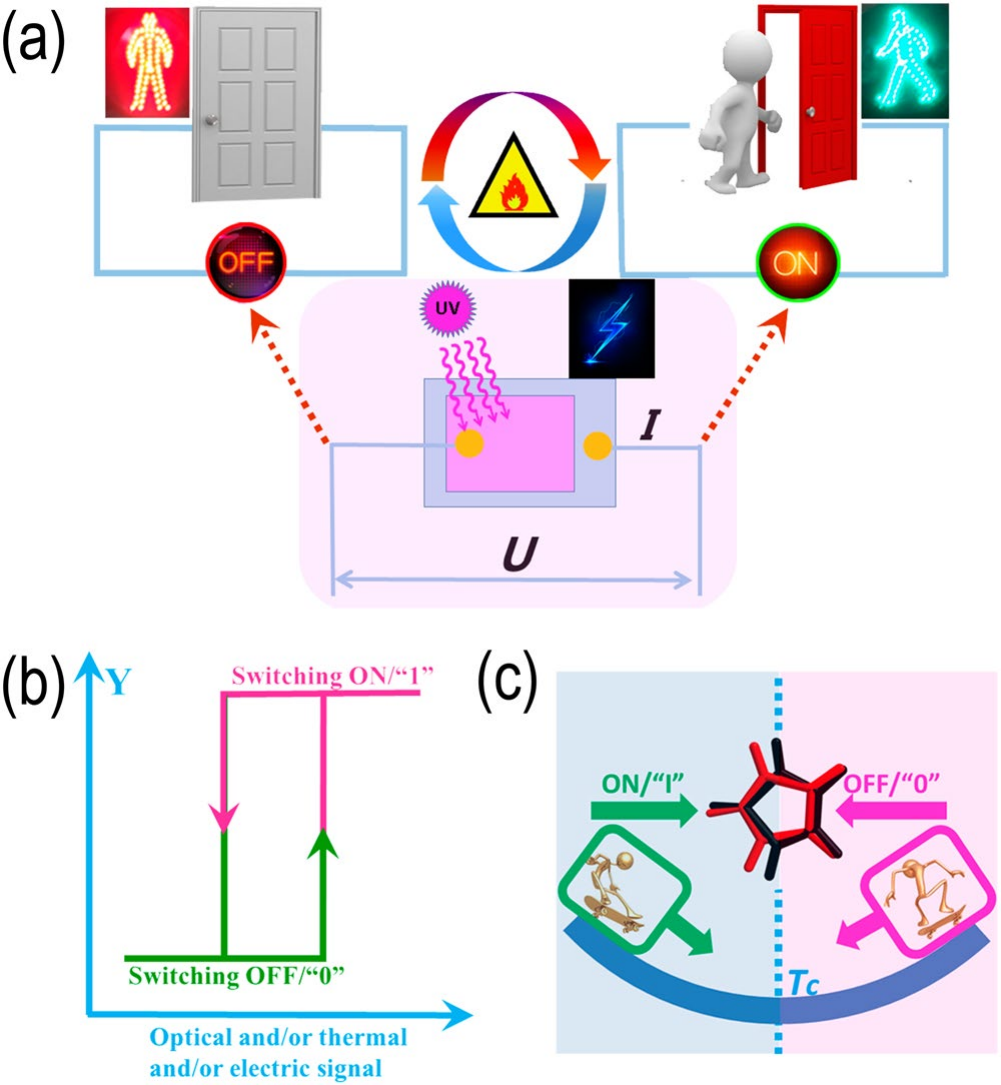
(**a**) Schematic of electric, optical, thermal triple controllable integrated Module. The optical and dielectric “ON/OFF” switches can be switched automatically and simultaneously, and controlled precisely by temperature or/and light wavelength signal. The two type switches can be combined into one sensitive synchronous responsive mode of photoelectric signal in the same temperature control system, which lays the foundation for the transformation of single channel and dual channel. (**b**) The schematic diagram of the bistable state of electric, optical, thermal signal switching. (**c**) HIm (α) cation exhibits interesting disordered motion similar to skateboarding, which arouses photoelectric multifunctional molecular switch to reach a switching ON/“1” or OFF/“0” states.

The material shows a very good thermal sensitivity, optical switching and dielectric switching effects, which can be integrated into one single-molecule module (as shown in Fig. 8). The triple controllable module was used to demonstrate the implement of switchable “ON/OFF” process using techniques of integrated flexible thin-film device. The entire switching process is fully automated according to different physical environment by combining effect of thermosensitive, photosensitive, and electroresponse integrated module. Meanwhile the module can perform single channel or dual channel selective response for different environmental stimuli, respectively. For example, different light wavelength stimulations contribute to intensity changes of the launch signal, and rapidly switching of signal strength can be implemented in the temperature control system. The different frequency of the electrical stimulation will produce different dielectric signals, and the signals can also be quickly switched in the temperature control system. The two type switches can be combined into one sensitive synchronous responsive mode of photoelectric signal in the same temperature control system, which lays the foundation for the transformation of single channel and dual channel. Furthermore, flexible thin film was successfully prepared by easy spin-coating approach. The unidirectional film plane along the a-axis is consistent with the photoelectric property test, which provides the feasibility for the preparation of integrated module devices. Thus, the compound 1 fully demonstrated the charactars as excellent photoelectric switching devices, and these qualities recommended it to increasingly prominent role.

## Conclusion

In summary, the perovskite-like intelligent controllable material ImMC with superior electric, optical, thermal triple controllable switching effect has been successfully synthesized. According to the microstructure and macroscopic properties analysis, every disordered α-type HIm cationic motion leads to a reversible photoelectric “ON”/“OFF” transform at ca.170 K. It is worth mentioning that flexible thin film was successfully prepared by low-cost spin-coating approach, which displays favorable unidirectionality and provides a basis for device applications. The dielectric bistability has confirmed that the crystal 1 possesses switch-ON/switch-OFF responses under different frequencies. After 325 nm wavelength excitation on block crystal, the emission spectra displays distinctive switch “ON”/“OFF” transform of peak intensity values between the RTP and the LTP. We are excited that compound 1 displays the excellent optical-electrical dual switches effect, and these superior performances will open a new avenue for the design of smart controllable switchable and sensing materials.

## Methods

### Preparation of crystals and thin films

All chemical reagents were commercially obtained (chemically pure) and used without further purification. The title complex 1 crystallized from an aqueous solution of MnCl_2_ (10 mmol), imidazole (30 mmol) and HCl (30 mmol) upon slow evaporation in 323 K Semi-opening oven. Yellow block crystals were obtained after several days. Single-layer thin films were fabricated on PET-ITO (PET: polyethylene terephthalate, ITO: Indium tin oxide) substrate by the simple spin-coating approach, with the rotary speed of 3000 rev/min (see Fig. 3 and the Supporting Information).The purity of the crystals was confirmed by IR spectroscopy (Figure S1, Supporting Information) and powder X-ray diffraction (PXRD) patterns of 1 at room temperature matched very well with the pattern simulated from the single crystal structure(Figure S2, Supporting Information).

### Single-crystal x-ray crystallography

Crystallographic data were collected by using a Rigaku Saturn 924 diffractometer with Mo-K*α* radiation (*λ* = 0.71073 Ǻ) at 123 K and 293 K. Data processing including empirical absorption correction was performed using the crystalclear software package (Rigaku, 2005). The structure was solved using direct methods and successive Fourier difference synthesis (SHELXS-2014), and refined using the full-matrix least-squares method on *F* ^2^ using the SHELXTL software package. All non-hydrogen atoms were located from the trial structure and refined anisotropically with SHELXTL using the fullmatrix least-squares procedure. The positions of hydrogen atoms were generated geometrically and allowed to ride on the parent atoms. Crystallographic data, details of the data collection and refinement are summarized in Table S1 (see Supporting Information).

### Measurement of physical properties

The electrodes were made by sputtering silver onto both sides of single-crystal specimens and attaching copper leads with silver paste. The temperature dependence of the dielectric constant was measured using a Tonghui TH2828A Precision LCR Meter in the temperature range of 100−300 K at the frequency of 1 MHz with the measuring AC voltage fixed at 1 V. The DSC measurement was performed on NETZCSCH DSC 200 F3 instrument by heating and cooling rate of 10 K/min in the temperature range of 100−300 K. A detailed method of Photoluminescence Measurements, SEM and AFM measurements can be seen in the Supporting Information.

## Electronic supplementary material

Supporting Information
