# Supplementary material for: Lead-free Single-molecule Switching Material with Electric, Optical, Thermal Triple Controllable Multifunction Based on Perovskite-like Crystal and Flexible Thin Film
Source: Sci Rep. 2017 Oct 2;7:12493. doi: 10.1038/s41598-017-12338-y (PMC5624872; doi:10.1038/s41598-017-12338-y)
Supplement: Supplementary file 1 — Supporting Information [file 41598_2017_12338_MOESM1_ESM.doc]

**Lead-free Single-molecule Switching Material with Electric, Optical, Thermal Triple Controllable Multifunction Based on Perovskite-like Crystal and Flexible Thin Film**

*Cheng Chen, Wan-Ying Zhang, Qiong Ye*, Da-Wei Fu**

**Experimental section**

**Thin-film Fabrication**

The crystal powders were dissolved in anhydrous methanol to form a saturated solution with a solubility of about 38%, and then the solution was filtered through a 0.22-um nylon membrane filter. With this solution, single-layer of thin film were deposited on ITO-PET substrate (PET: polyethylene terephthalate, ITO: indium tin oxide) by the spin coating method, with the rotary speed of 3000 rev/min. After that, a single-crystal-like thin film is formed. Finally, the grown film was dried and annealed at 60°C for 30 min.

**Photoluminescence Measurements**

A HORIBA FluoroMax-4 fluorescence photometer was used for fluorescence powders sample measurements (excitation spectra and emission spectra). Yellow single-crystal samples of **1** were used for the temperature dependence of photoluminescence measurements on a LabRAM HR800 Raman system with an excitation wavelength of 325 nm in the temperature range from 100 to 273 K. The laser spot size on the samples was about 1 μm with a 50× objective.

**SEM and AFM measurements**

A film of about 0.4 * 0.6 cm^2^ size is cut from the sample 1 and glued to the electrode for SEM testing. The surface morphologies of thin film (Figure S5) were observed by SEM (Phenom ProX), and the thickness was measured to be about 2-10 μm. To observe the thin-film morphology of 1 by atomic force microscopy (AFM), the whole thin-film sample deposited on PET-ITO can be fixed on the AFM sample holder. The images were collected by Asylum Research MFP-3D and Brucker Multimode 8 systems, using a conductive cantilever with a spring constant of 2 Nm^-1^.all the zoomed areas were scanned only once to collect the possible intrinsic micro-structural images.

**
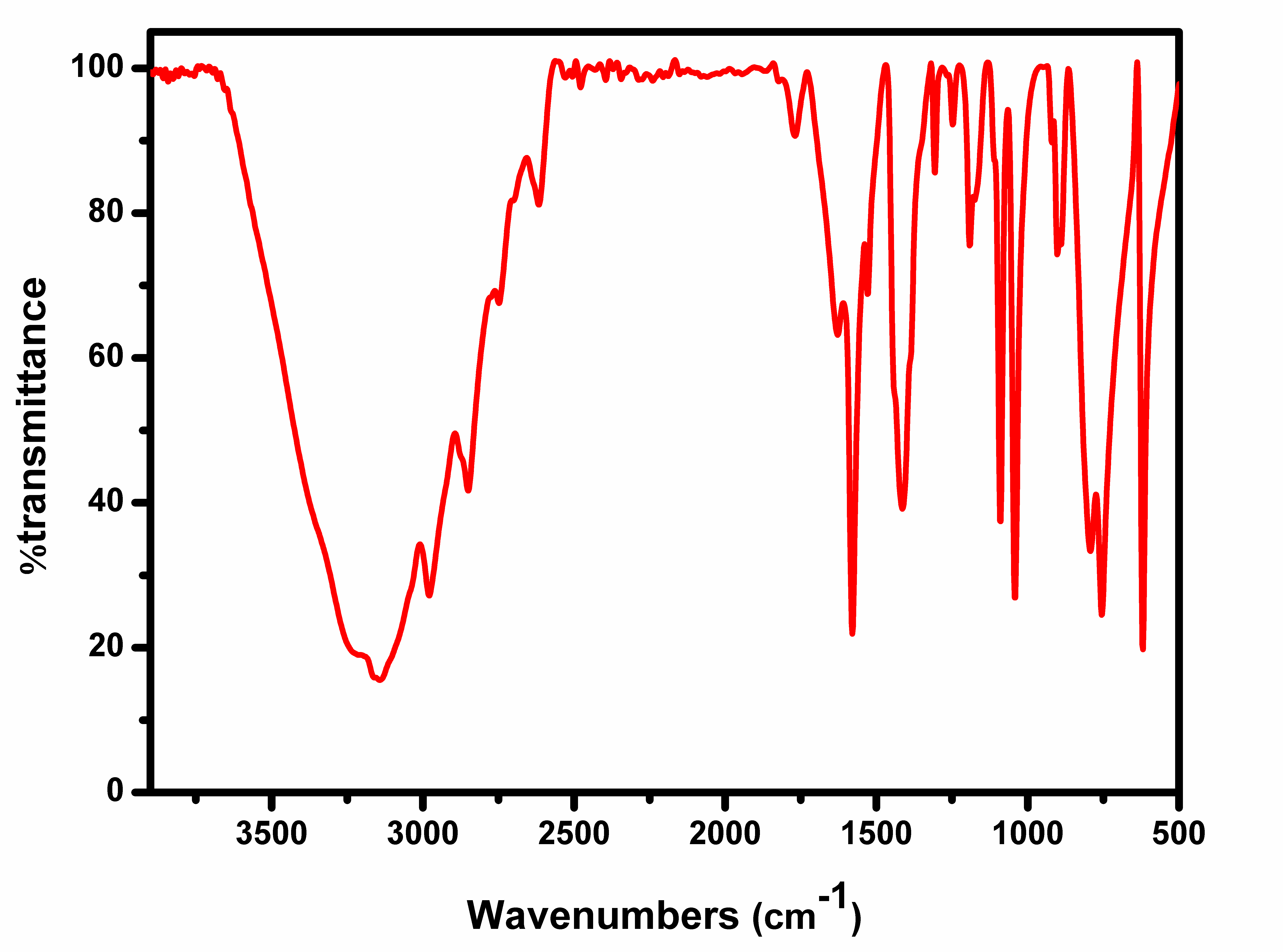
**

Figure S1. The IR spectrum of compound 1 shows evident characteristic peaks near ~3000 cm^-1^, ~1500 cm^-1^, ~1000 cm^-1^ and ~700 cm^-1^, which is consistent with imidazole standard atlas.

**
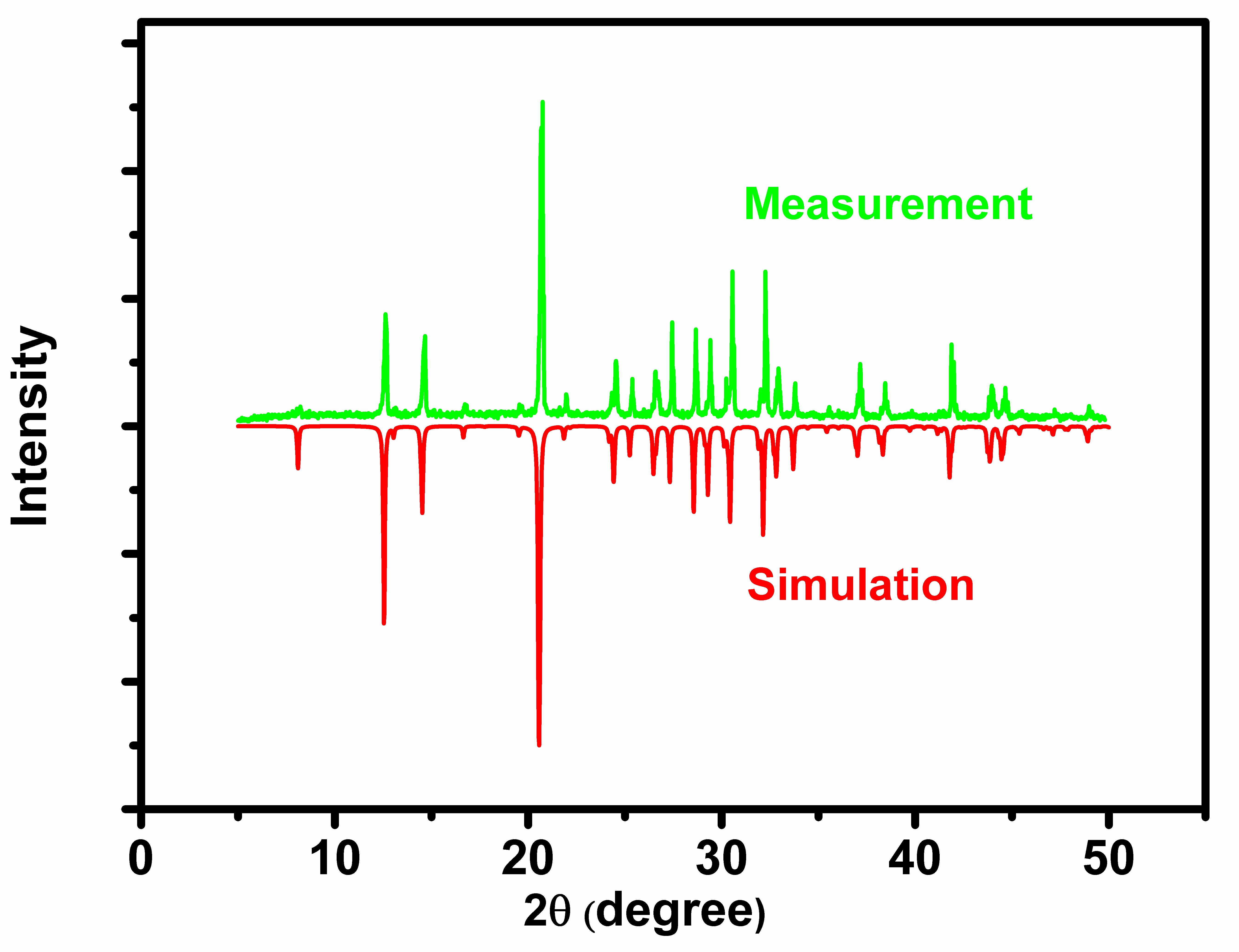
**

Figure S2. The powder X-ray diffraction (PXRD) patterns of 1 at room temperature matched very well with the pattern simulated from the single crystal structure.

**
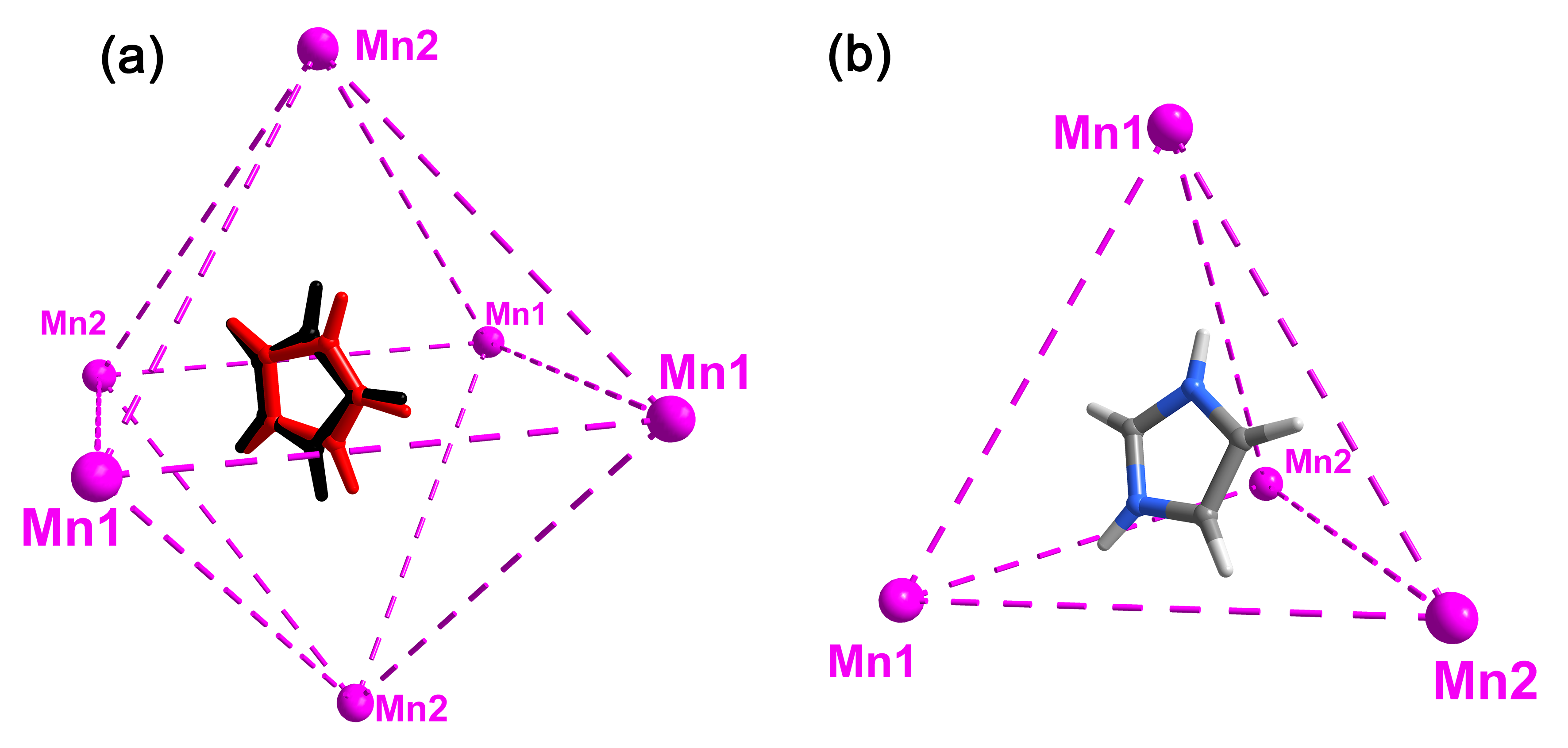
**

Figure S3. The HIm(α) cations display disordered states in an Octahedral space (a) when the HIm (β) cations keep static state in a narrow tetrahedron space (b) in the room temperature, indicating that special space environment offer moving possibility of HIm(α) cations in compound 1. (Mn1 on behalf of octahedral (MnCl_6_)^4−^ anions and Mn2 on behalf of tetrahedral (MnCl_4_)^2−^ anions)

**
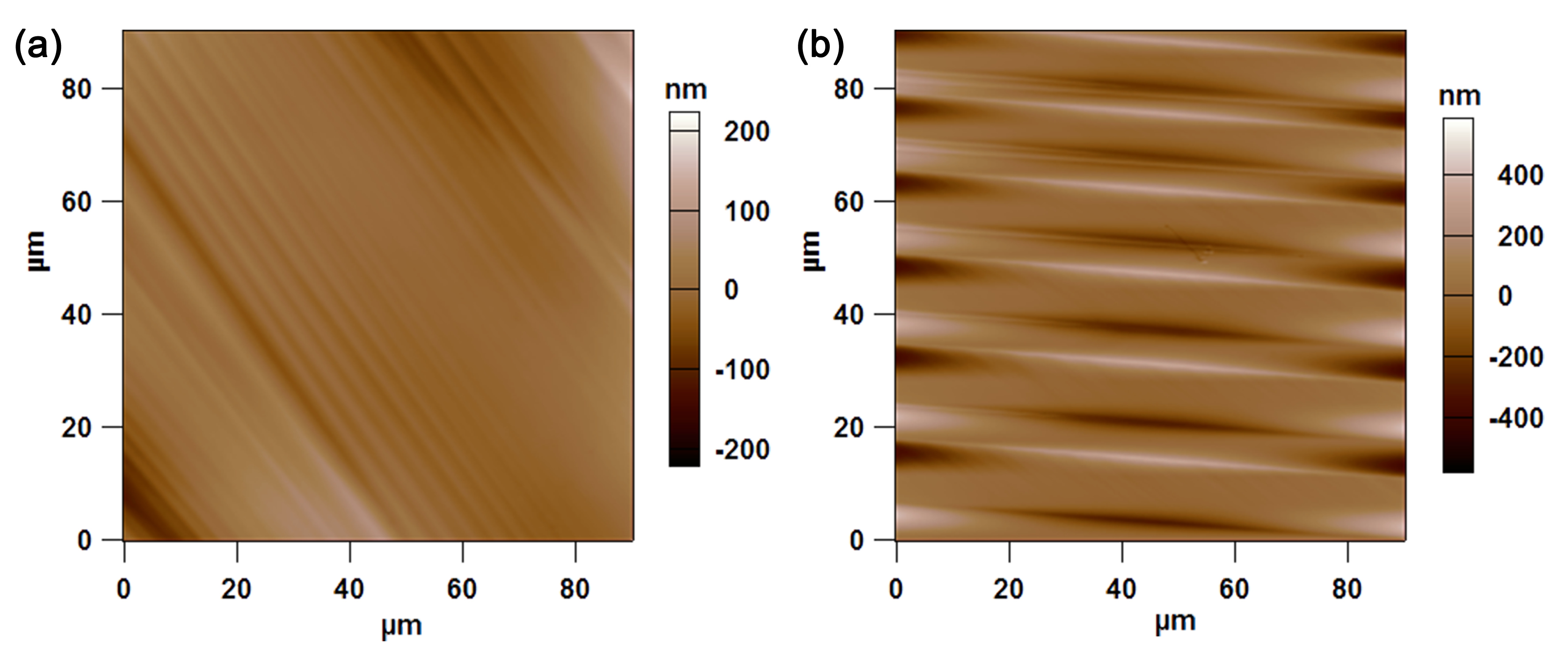
**

Figure S4. Morphologies of the smooth (a) and fluctuate (b) regions of the (HIm)_6_•[MnCl_4_•MnCl_6_] thin film on a 90*90 μm^2^ scale on ITO substrate. The film is prepared by spreading a thin layer saturated aqueous solution on substrate plane to promote crystal growth, and a single-crystal-like thin film is formed after annealing process at 80℃ for 1 hour. The thickness of (HIm)6•[MnCl4•MnCl6] film is about 2 mm through measurement of a man-made gap with AFM, which is 100 times of thin film prepared by spin-coating method.


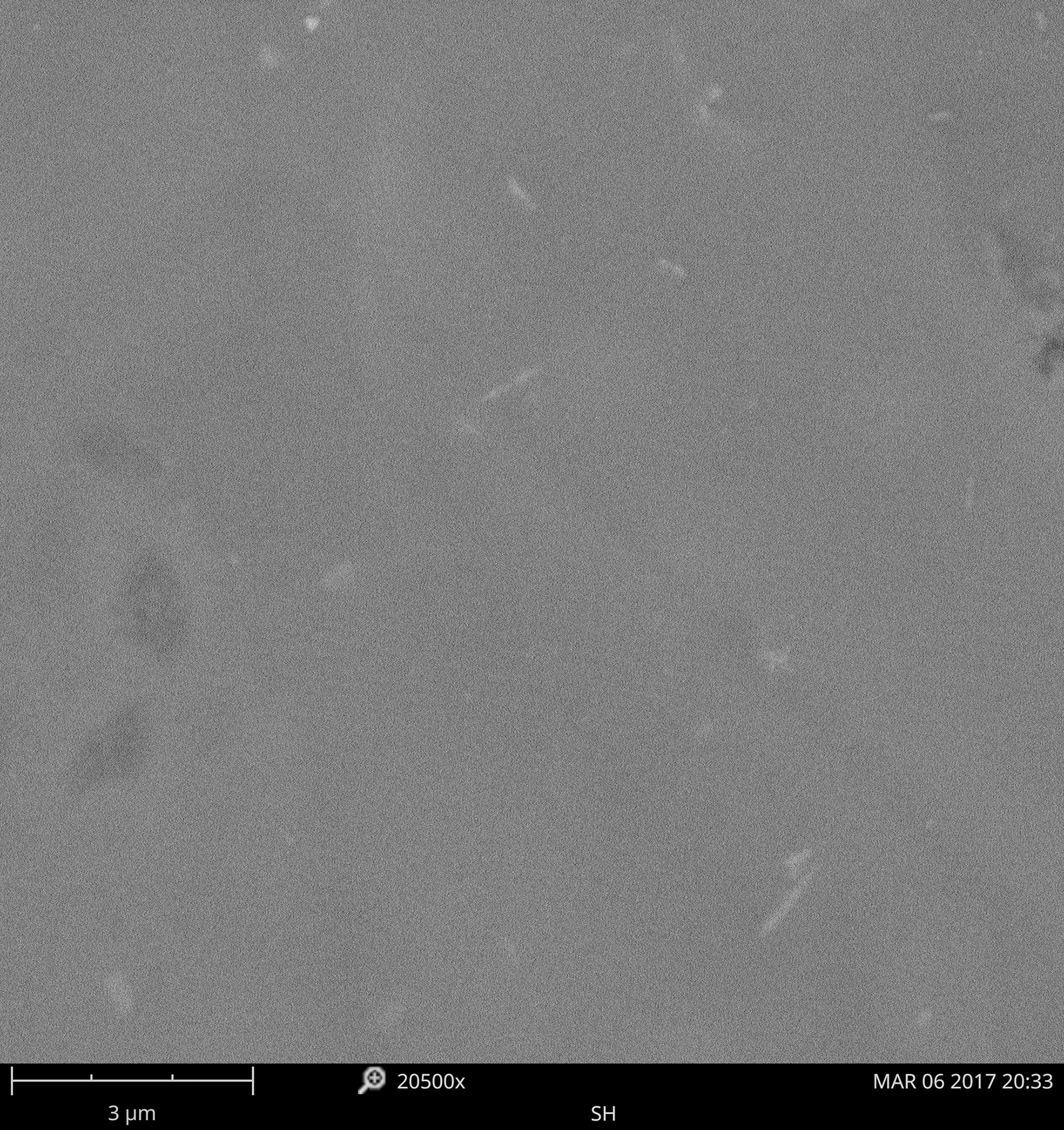


Figure S5. Scanning electron microscopy (SEM) topography displaying the dense and uniform surface of single-layer (HIm)_6_•[MnCl_4_•MnCl_6_] crystal thin film with dimension on the micrometer scale.


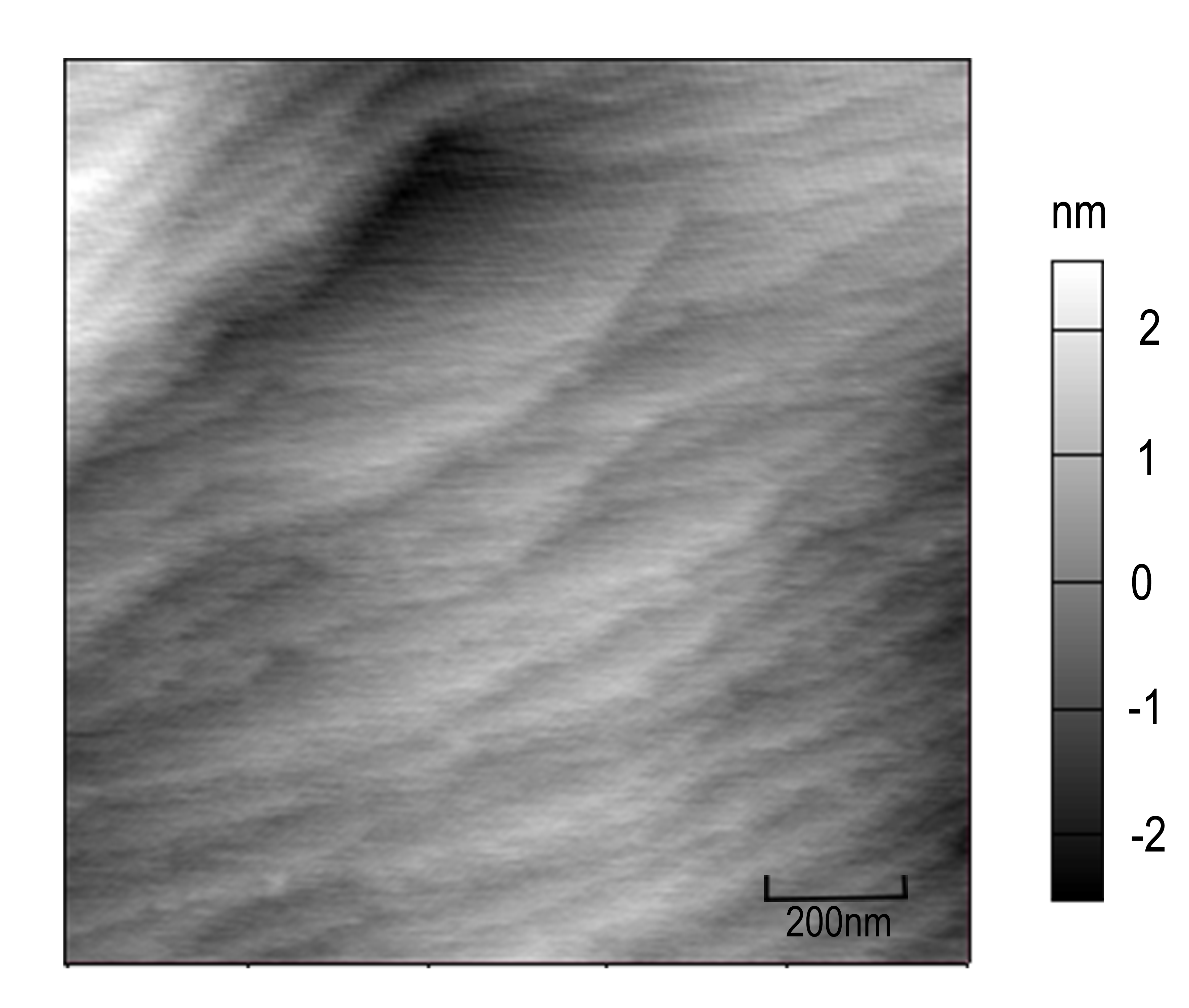


Figure S6. Atomic force microscopy (AFM) image (~200nm) shows well crystallized and homogeneously distributed of the grains in the thin-film surface.


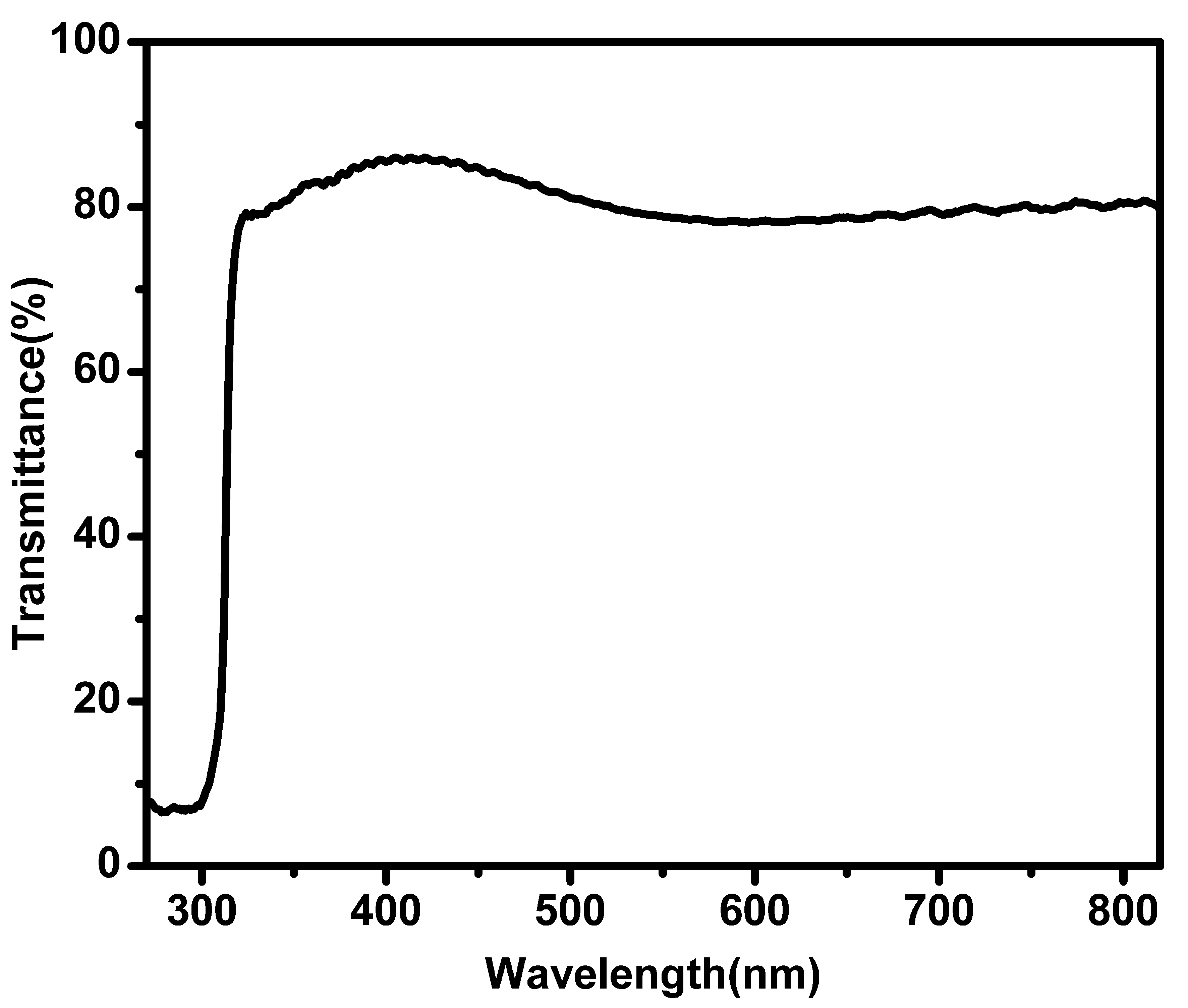


Figure S7. Optical transmission spectrum of the ImMC thin film displays a transmission of 80% or more in visible region.

**Table S1**  Crystal data and structure refinements for **1** at 123 K and 293 K.

|  | LTP (123 K) HTP (293 K) | |
| --- | --- | --- |
| Empirical formula  Formula weight | [C_3_N_2_H_5_]_6_·[MnCl_4_·MnCl_6_]  878.92 | [C_3_N_2_H_5_]_6_·[MnCl_4_·MnCl_6_]  878.92 |
| Crystal system | Tetragonal | Tetragonal |
| Space group | *I4(1)/a* | *I4(1)/a* |
| *a*/Å | 12.028(4) | 12.1879(17) |
| *b*/Å | 12.028(4) | 12.1879(17) |
| *c*/Å | 24.471(10) | 24.504(5) |
| Volume (Å^3^) | 3540(3) | 3639.9(13) |
| *F*(000) | 1768 | 1768 |
| Collected reflections | 7680 | 12550 |
| Unique reflections | 1541 | 2091 |
| Absorption correction | Multi-scan | Multi-scan |
| Z, Calculated density  Goodness-of-fit on F^2^ | 4, 1.142 Mg/m^3^  1.137 | 4, 1.604 Mg/m^3^  1.136 |
| *R*_1_/*wR*_2_ [ *I>2σ( I )*] | 0.0534/ 0.1590 | 0.0310/ 0.0597 |

**Table S2 Selected structural data for 1 under 293 K**

| *Bond lengths / Å and bond angles / °* |
| --- |
| Mn(1)-Cl(2) 2.5505(4) Mn(1)-Cl(2)#1 2.5506(4))  Mn(1)-Cl(2)#2 2.5506(4) Mn(1)-Cl(2)#3 2.5506(4)  Mn(1)-Cl(1)#3 2.6050(7) Mn(1)-Cl(1) 2.6051(7)  Mn(2)-Cl(3) 2.3584(5) Mn(2)-Cl(3)#4 2.3584(5)  Mn(2)-Cl(3)#5 2.3584(5) Mn(2)-Cl(3)#6 2.3584(5)  N(1)-C(1) 1.2906(19) N(1)-C(2) 1.339(2)  N(2)-C(1) 1.2883(19) N(2)-C(3) 1.340(2)  C(2)-C(3) 1.316(2) C(4)-N(3) 1.454(4)  C(4)-N(3)#7 1.454(4) C(5)-C(5)#7 1.080(3)  C(5)-N(3) 1.232(3) C(5')-C(5')#7 1.300(3)  C(4')-N(3') 1.110(5) C(4')-N(3')#7 1.110(5)  N(3')-C(5') 1.424(3) |

Symmetry transformations used to generate equivalent atoms:

#1 -y+1/4,x+1/4,-z+1/4 #2 -x+0,-y+1/2,z+0 #3 y-1/4,-x+1/4,-z+1/4

#4 -y+5/4,x+1/4,-z+1/4 #5 -x+1,-y+3/2,z+0 #6 y-1/4,-x+5/4,-z+1/4

#7 -x+0,-y+3/2,z+0

**Table S3 Selected structural data for 1 under 123 K**

| *Bond lengths / Å and bond angles / °* |
| --- |
| Mn(1)-Cl(2)#1 2.5402(13) Mn(1)-Cl(2)#2 2.5402(13)  Mn(1)-Cl(2)#3 2.5402(13) Mn(1)-Cl(2) 2.5402(13)  Mn(1)-Cl(1) 2.5927(18) Mn(1)-Cl(1)#3 2.5927(18)  Mn(2)-Cl(3) 2.3625(13) Mn(2)-Cl(3)#4 2.3625(13)  Mn(2)-Cl(3)#5 2.3625(13) Mn(2)-Cl(3)#6 2.3625(13)    N(1)-C(3) 1.310(6) N(1)-C(2) 1.361(7) N(2)-C(3) 1.318(6) N(2)-C(1) 1.357(7)  N(3)-C(4) 1.335(7) N(3)-C(5) 1.355(9)  C(1)-C(2) 1.341(7) C(4)-N(3)#7 1.335(7)  C(5)-C(5)#7 1.286(14) |

Symmetry transformations used to generate equivalent atoms:

#1 -x+2,-y+3/2,z+0 #2 -y+7/4,x-1/4,-z+7/4 #3 y+1/4,-x+7/4,-z+7/4

#4 y+1/4,-x+3/4,-z+7/4 #5 -x+1,-y+1/2,z+0 #6 -y+3/4,x-1/4,-z+7/4

#7 -x+2,-y+1/2,z+0
